
JOURNAL INFORMATION
==============================
NLM Title Abbreviation: Hum Brain Mapp
Journal ID: Hum Brain Mapp
NLM Journal ID: 9419065
Journal ID: HBM

Human Brain Mapping

ISSN: 1065-9471
EISSN: 1097-0193
Publisher: Wiley

ARTICLE INFORMATION
==============================
PMCID: PMC11474869
DOI: 10.1002/hbm.25942
Article ID: HBM25942
Article version: 1
Subjects: Issue Information, Issue Information

Editorial board ‐ TOC

Electronic publication date: 2023 Nov 27
Publication date: 2023 Dec 15
Volume: 44
Issue: 18
First page: 6289
Last page: 6292
Copyright: © 2023 The Authors. Human Brain Mapping published by Wiley Periodicals LLC.
License: This is an open access article under the terms of the http://creativecommons.org/licenses/by/4.0/ License, which permits use, distribution and reproduction in any medium, provided the original work is properly cited.
License URL: https://creativecommons.org/licenses/by/4.0/

Figure count: 0
Table count: 0
Page count: 6

source-schema-version-number: 2.0
cover-date: 15 December, 2023
details-of-publishers-convertor: Converter:WILEY_ML3GV2_TO_JATSPMC version:6.4.9 mode:remove_FC converted:15.10.2024

==============================

### HUMAN BRAIN MAPPING

Open Access

Volume 44, Issue 18, 15 December, 2023

View this journal online at wileyonlinelibrary.com

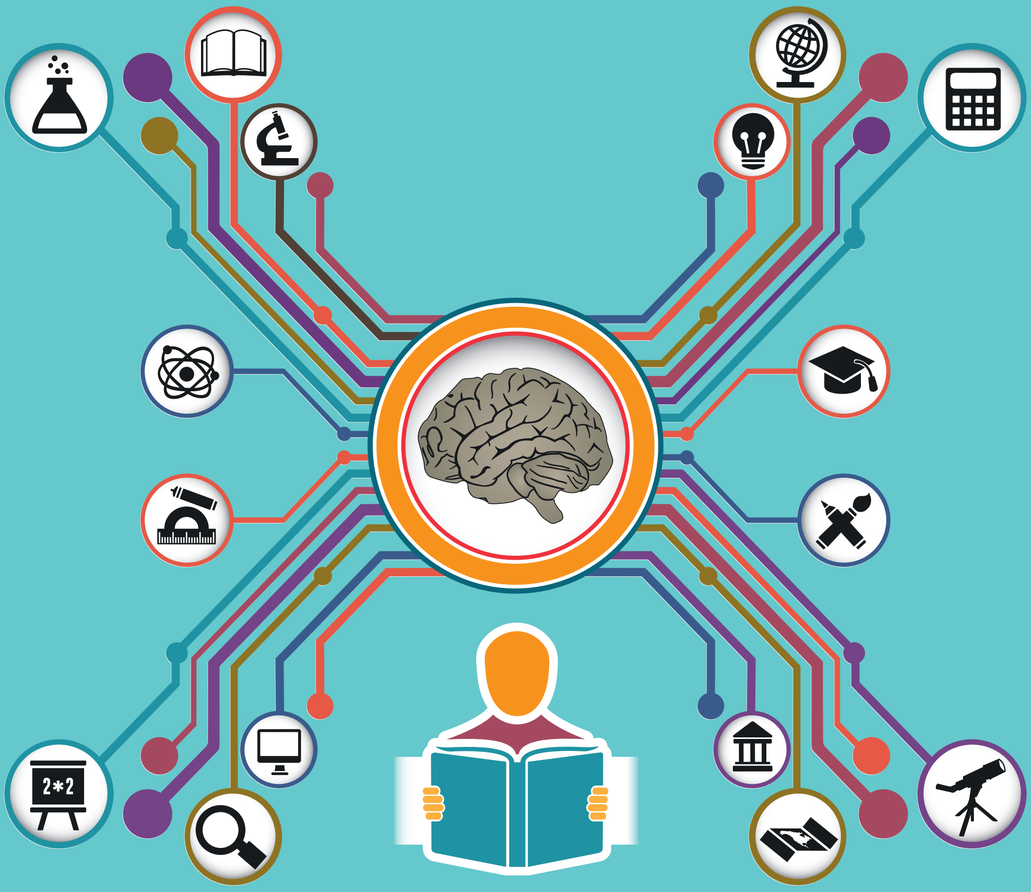

WILEY

ISSN 1097-0193

### HUMAN BRAIN MAPPING

### A Journal Devoted to Functional Neuroanatomy and Neuroimaging

### *Editor-in-Chief*

**Simon B. Eickhoff**
Institute for Systems Neuroscience
Heinrich-Heine University, Düsseldorf, Germany

### *Deputy Editors-in-Chief*

**Gary Egan**
Monash Biomedical Imaging
Monash University, Victoria, Australia

**Sophia Frangou**
Icahn School of Medicine
at Mount Sinai, New York, USA

**Martijn van den Heuvel**
Brain Center Rudolf Magnus,
University of Medical Center,
Utrecht, Netherlands

### *Associate Editors*

**Annabel Chen**
Nanyang Technological University, Singapore

**Michael J. Farrell**
Monash University, Victoria, Australia

**Massimo Filippi**
Università Vita-Salute San Raffaele, Milan,
Italy

**Sophia Frangou**
Icahn School of Medicine at Mount Sinai
New York, USA

**Cheryl L. Grady**
Rotman Research Institute
Toronto, Canada

**Joachim Gross**
Centre for Cognitive Neuroimaging
University of Glasgow, Glasgow, UK

**Martin A. Lindquist**
John Hopkins, Bloomberg School of Public
Health, MD, USA

**Tomáš Paus**
Rotman Research Institute
Toronto, Canada

**Indira Tendolkar**
Radboud University, Nijmegen,
The Netherlands

**Paul Thompson**
Laboratory of Neuro Imaging,
University of Southern California,
Los Angeles, CA, USA

**Helen Zhou**
Yong Loo Lin School of Medicine, National
University of Singapore, Singapore

### *Editorial Board*

**Susan Bookheimer**
Center for Cognitive Neuroscience
UCLA School of Medicine, USA

**Allen R. Braun**
NIDCD, National Institutes of Health
Bethesda, USA

**Christian Buechel**
Univ-Krankenhaus Eppendorf
Hamburg, Germany

**Marco Catani**
King's College London, UK

**Franco Cauda**
University of Turin, Italy

**Vincent Clark**
University of New Mexico, USA

**Olivier David**
Université Joseph Fourier, France

**Mark D'Esposito**
University of California, Berkeley,
USA

**Julien Doyon**
Functional Neuroimaging Unit
University of Montreal, Canada

**Eugene Duff**
University of Oxford, UK

**Timothy Q. Duong**
Research Imaging Institute,
University of Texas Health Science
Center, USA

**Paul Fletcher**
University of Cambridge, UK

**Jia-Hong Gao**
Brain Research Imaging
Center The University of Chicago,
USA

**David C. Glahn**
Yale University School of Medicine
Hartford, USA

**Gregory S. Hickok**
Center for Cognitive
Neuroscience University of California,
Irvine, USA

**Barry Horwitz**
NIDCD, National Institutes of Health
USA

**Peter Jezzard**
John Radcliffe Hospital
University of Oxford, UK

**David N. Kennedy**
Harvard Medical School, USA

**Neda Ladbon-Bernasconi**
Montreal Neurological Institute
Canada

**Nicholas Lange**
Harvard Medical School, USA

**Tatia M. C. Lee**
State Key Laboratory of Brain &
Cognitive Sciences, The University of
Hong Kong

**Louis Lemieux**
UCL Institute of Neurology, UK

**Bernard Mazoyer**
CNRS/CEA, Université de Bordeaux
France

**Gregory McCarthy**
Yale University, USA

**A. Randy McIntosh**
Rotman Research Institute
Toronto, Canada

**Janine Mendola**
McGill Vision Research
McGill University, Canada

**Thomas Nichols**
University of Warwick, UK

**William Penny**
Wellcome Trust Centre for
Neuroimaging, UCL Institute of
Neurology, UK

**Laurent Petit**
CNRS, Université de Bordeaux,
France

**Russel A. Poldrack**
Stanford Unversity, USA

**Jean-Baptiste Poline**
Imagen Orsay Service Hospitalier
Frédéric Joliot, France

**Stephen Smith**
Center for Functional MRI of the
Brain, University of Oxford, UK

**Elizabeth Sowell**
Children'sHospital Los Angeles, USA

**Li Hai Tan**
Shenzhen Institute of Neuroscience
Shenzhen, P.R. China

**Kate Watkins**
John Radcliffe Hospital
University of Oxford, UK

**Richard G. Wise**
Cardiff University, UK

**Roger Woods**
UCLA School of Medicine, USA

**Mark Woolrich**
Oxford Centre for Human Brain
Activity, University of Oxford, UK

**Jinhu Xiong**
University of Iowa, USA

**Robert Zatorre**
MontrealNeurological Institute, Canada

**Open Access and Copyright**
All articles published by *Human Brain Mapping* submitted after January 1, 2020 are fully open access: immediately freely available to read, download, and share. All articles accepted from August 6, 2019 are published under the terms of a Creative Commons license.
Copyright on any research article published by *Human Brain Mapping* is retained by the author(s).

Further information about open access licenses and copyright can be found at: https://authorservices.wiley.com/open-research/open-access/index.html. Information on the licenses used by *Human Brain Mapping* can be found at: https://onlinelibrary.wiley.com/journal/10970193.

**Disclaimer**
The Publisher and Editors cannot be held responsible for errors or any consequences arising from the use of information contained in this journal; the views and opinions expressed do not necessarily reflect those of the Publisher and Editors, neither does the publication of advertisements constitute any endorsement by the Publisher and Editors of the products advertised. Wiley Open Access articles posted to repositories or websites are without warranty from Wiley of any kind, either express or implied, including, but not limited to, warranties of merchantability, fitness for a particular purpose, or non-infringement. To the fullest extent permitted by law Wiley disclaims all liability for any loss or damage arising out of, or in connection, with the use of or inability to use the content.

HUMAN BRAIN MAPPING

A Journal Devoted to
Functional Neuroanatomy
and Neuroimaging

VOLUME 44, NUMBER 18, 15 DECEMBER 2023

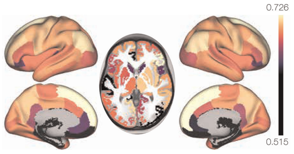

*See article by, Capouskova,et al., pages 6349–6363, this issue*

### CONTENTS

◆ *6293* Functional brain connectivity predicts sleep duration in youth and adults
**Anurima Mummaneni, Omid Kardan, Andrew J. Stier, Taylor A. Chamberlain, Alfred F. Chao, Marc G. Berman, and Monica D. Rosenberg**
***Published online 2 November 2023***

◆ *6308* Developmental dyscalculia is not associated with atypical brain activation: A univariate fMRI study of arithmetic, magnitude processing, and visuospatial working memory
**Fu Yu Kwok, Eric D. Wilkey, Lien Peters, Ellyn Khiu, Rebecca Bull, Kerry Lee, and Daniel Ansari**
***Published online 1 November 2023***

◆ *6326* Bayesian longitudinal tensor response regression for modeling neuroplasticity
**Suprateek Kundu, Alec Reinhardt, Serena Song, Joo Han, M. Lawson Meadows, Bruce Crosson, and Venkatagiri Krishnamurthy**
***Published online 1 November 2023***

◆ *6349* Integration and segregation manifolds in the brain ensure cognitive flexibility during tasks and rest
**Katerina Capouskova, Gorka Zamora-López, Morten L. Kringelbach, and Gustavo Deco**
***Published online 17 October 2023***

◆ *6364* Morphological connectivity differences in Alzheimer's disease correlate with gene transcription and cell-type
**Huiying Yu, Yanhui Ding, Yongbin Wei, Martin Dyrba, Dong Wang, Xiaopeng Kang, Weizhi Xu, Kun Zhao, and Yong Liu, for the Alzheimer's Disease Neuroimaging Initiative**
***Published online 17 October 2023***

(*continued on overleaf*)

(*continued from overleaf*)

◆ *6375* T1 and FLAIR signal intensities are related to tau pathology in dominantly inherited Alzheimer disease
**Farzaneh Rahmani, Matthew R. Brier, Brian A. Gordon, Nicole McKay, Shaney Flores, Sarah Keefe, Russ Hornbeck, Beau Ances, Nelly Joseph-Mathurin, Chengjie Xiong, Guoqiao Wang, Cyrus A. Raji, Jorge J. Libre-Guerra, Richard J. Perrin, Eric McDade, Alisha Daniels, Celeste Karch, Gregory S. Day, Adam M. Brickman, Michael Fulham, Clifford R. Jack Jr, Christian la La Fougère, Gerald Reischl, Peter R. Schofield, Hwamee Oh, Johannes Levin, Jonathan Vöglein, David M. Cash, Igor Yakushev, Takeshi Ikeuchi, William E. Klunk, John C. Morris, Randall J. Bateman, and Tammie L. S. Benzinger, For the Dominantly Inherited Alzheimer Network (DIAN)**
***Published online 23 October 2023***

◆ *6388* Dehydroepiandrosterone mediates associations between trauma-related symptoms and anterior pituitary volume in children and adolescents
**Giorgia Picci, Chloe C. Casagrande, Lauren R. Ott, Nathan M. Petro, Nicholas J. Christopher-Hayes, Hallie J. Johnson, Madelyn P. Willett, Hannah J. Okelberry, Yu-Ping Wang, Julia M. Stephen, Vince D. Calhoun, and Tony W. Wilson**
***Published online 18 October 2023***

◆ *6399* Test–retest reliability and predictive utility of a macroscale principal functional connectivity gradient
**Annchen R. Knodt, Maxwell L. Elliott, Ethan T. Whitman, Alex Winn, Angela Addae, David Ireland, Richie Poulton, Sandhya Ramrakha, Avshalom Caspi, Terrie E. Moffitt, and Ahmad R. Hariri**
***Published online 18 October 2023***

◆ *6418* Revisiting deficits in threat and safety appraisal in obsessive-compulsive disorder
**Luke J. Hearne, Michael Breakspear, Ben J. Harrison, Caitlin V. Hall, Hannah S. Savage, Conor Robinson, Saurabh Sonkusare, Emma Savage, Zoie Nott, Leo Marcus, Sebastien Naze, Bjorn Burgher, Andrew Zalesky, and Luca Cocchi**
***Published online 18 October 2023***

◆ *6429* Changes in aspartate metabolism in the medial-prefrontal cortex of nicotine addicts based on *J*-edited magnetic resonance spectroscopy
**Miaomiao Yu, Liangjie Lin, Ke Xu, Man Xu, Jianxin Ren, Xiaoyu Niu, Xinyu Gao, Mengzhe Zhang, Zhengui Yang, Jinghan Dang, Qiuying Tao, Shaoqiang Han, Weijian Wang, Jingliang Cheng, and Yong Zhang**
***Published online 1 November 2023***

(*continued on overleaf*)

(*continued from overleaf*)

◆ *6439* Separate and overlapping mechanisms of statistical regularities and salience processing in the occipital cortex and dorsal attention network
**Bertrand Beffara, Fadila Hadj-Bouziane, Suliann Ben Hamed, C. Nico Boehler, Leonardo Chelazzi, Elisa Santandrea, and Emiliano Macaluso**
***Published online 24 October 2023***

◆ *6459* Lifelong olfactory deprivation-dependent cortical reorganization restricted to orbitofrontal cortex
**Moa G. Peter, Fahimeh Darki, Evelina Thunell, Gustav Mårtensson, Elbrich M. Postma, Sanne Boesveldt, Eric Westman, and Johan N. Lundström**
***Published online 2 November 2023***

◆ *6471* Association between activity in the ventral premotor cortex and spinal cord activation during force generation—A combined cortico-spinal fMRI study
**Hanna Braaß, Jan Feldheim, Ying Chu, Alexandra Tinnermann, Jürgen Finsterbusch, Christian Büchel, Robert Schulz, and Christian Gerloff**
***Published online 24 October 2023***

◆ *6484* EEG microstates in early-to-middle childhood show associations with age, biological sex, and alpha power
**Aron T. Hill, Neil W. Bailey, Reza Zomorrodi, Itay Hadas, Melissa Kirkovski, Sushmit Das, Jarrad A. G. Lum, and Peter G. Enticott**
***Published online 24 October 2023***

◆ *6499* Treating amblyopia using altered reality enhances the fine-scale functional correlations in early visual areas
**Xue Dong, Lijuan Liu, Xinxin Du, Yue Wang, Peng Zhang, Zhihao Li, and Min Bao**
***Published online 6 November 2023***

◆ *6511* Regular cannabis use alters the neural dynamics serving complex motor control
**Thomas W. Ward, Seth D. Springer, Mikki Schantell, Jason A. John, Lucy K. Horne, Anna T. Coutant, Hannah J. Okelberry, Madelyn P. Willett, Hallie J. Johnson, Abraham D. Killanin, Elizabeth Heinrichs-Graham, and Tony W. Wilson**
***Published online 13 November 2023***

◆ *6523* Neuroplastic changes in functional wiring in sensory cortices of the congenitally deaf: A network analysis
**Michaela Ruttorf, Zohar Tal, Lénia Amaral, Fang Fang, Yanchao Bi, and Jorge Almeida**
***Published online 13 November 2023***

(*continued on overleaf*)

(*continued from overleaf*)

◆ *6537* Characterizing systemic physiological effects on the blood oxygen level dependent signal of resting-state fMRI in time-frequency space using wavelets
**Quimby N. Lee, Jingyuan E. Chen, Gregory J. Wheeler, and Audrey P. Fan**
***Published online 11 November 2023***

◆ *6552* Brain dopamine receptor system is not altered in obesity: Bayesian and frequentist meta-analyses
**Kyoungjune Pak and Lauri Nummenmaa**
***Published online 11 November 2023***

◆ *6561* Predictive modelling of brain disorders with magnetic resonance imaging: A systematic review of modelling practices, transparency, and interpretability in the use of convolutional neural networks
**Shane O'Connell, Dara M. Cannon, and Pilib Ó. Broin**
***Published online 1 November 2023***

◆ *6575* Estimating the viscoelastic properties of the human brain at 7 T MRI using intrinsic MRE and nonlinear inversion
**Marius Burman Ingeberg, Elijah Van Houten, and Jaco J. M. Zwanenburg**
***Published online 1 November 2023***
